# Novel Fibrinogen- and Procalcitonin-Based Composite Ratios for Mortality Prediction in Pediatric Trauma

**DOI:** 10.3390/children13101288

**Published:** 2026-09-22

**Authors:** Fatih Durak, Emine Pinar Kulluoglu, Sevgi Sakman, Gokcen Ozcifci

**Affiliations:** 1 Department of Pediatric Intensive Care Unit, Izmir Katip Celebi University Medical Faculty, Izmir 35030, Turkey; fatih.durak@ikc.edu.tr; 2 Department of Pediatric Intensive Care Unit, Izmir City Hospital, Izmir 35030, Turkey; 3 Department of Pediatrics, Izmir City Hospital, Izmir 35030, Turkey; sevgi.sakman@saglik.gov.tr

**Keywords:** pediatric trauma, lactate/fibrinogen ratio, procalcitonin/albumin ratio, lethal triad, intensive care unit, mortality, biomarker kinetics

## Abstract

**Highlights:**

**What are the main findings?**

- The novel lactate/fibrinogen ratio (LFR) achieved the highest discriminatory performance for in-hospital mortality among all biomarkers tested [area under the receiver operating characteristic curve (AUC) 0.848], performing comparably to the established lactate/albumin ratio (LAR) despite reflecting a distinct physiological process—coagulopathy rather than generalized reserve.
- Using bootstrap-optimism-corrected analysis and a sensitivity analysis in an unrestricted cohort that additionally included early deaths and rapidly discharged survivors, only LAR and LFR showed consistently robust, timing-independent discrimination. The discriminatory performance of the procalcitonin/albumin ratio (PAR), the composite index TIPI, and the procalcitonin/fibrinogen ratio (PFR) was not robust to this test, and serial biomarker trajectories provided only limited, largely inconclusive additional prognostic value.

**What are the implications of the main findings?**

- Fibrinogen may serve as a viable alternative to albumin for normalizing lactate-based composite biomarkers in pediatric trauma, supporting a novel, coagulopathy-focused prognostic framework grounded in the classic “lethal triad” concept.
- The discriminatory value of inflammation-based ratios (PAR, TIPI, PFR) may be specific to patients surviving beyond the earliest, most rapidly fatal phase of injury, whereas lactate- and fibrinogen-based ratios (LAR, LFR) appear robust across the full spectrum of injury timing and severity—a distinction that should inform how these biomarkers are validated and applied in future, larger studies.

**Abstract:**

Background: Composite biomarkers combining inflammatory or metabolic markers with a negative acute-phase protein predict outcomes in critically ill patients, but fibrinogen-based ratios grounded in the “lethal triad” of trauma-induced coagulopathy remain unexplored in pediatric trauma. We evaluated novel lactate/fibrinogen (LFR) and procalcitonin/fibrinogen (PFR) ratios alongside the procalcitonin/albumin ratio (PAR), a composite triple inflammation–perfusion index (TIPI, combining procalcitonin, CRP, and lactate relative to albumin), and established albumin-based ratios and severity scores for mortality prediction. Methods: In this retrospective cohort study, 200 pediatric trauma patients admitted to the PICU of İzmir City Hospital (27 October 2023–27 October 2025) were analyzed. All ratios were calculated serially over the first 72 h and at PICU discharge. Discrimination was assessed by ROC analysis. Independent predictors were identified using Firth penalized logistic regression, with bootstrap validation (B = 1000). Results: Thirteen patients (6.5%) died. LFR achieved the highest point-estimate discrimination (AUC 0.848), numerically exceeding LAR (0.829), though not significantly (*p* = 0.399). PAR, TIPI, and PFR were also independently associated with mortality (all *p* < 0.05), whereas CAR and NLR were not. LFR correlated strongly with LAR (ρ = 0.873), indicating overlapping information despite differing denominators. Serial trajectories provided only limited, largely inconclusive incremental value. A sensitivity analysis including early deaths and rapid discharges (*n* = 274) confirmed LFR/LAR’s robust discrimination. but not that of PAR, TIPI, or PFR. Conclusions: Lactate-based ratios showed the strongest single-timepoint discrimination, with the novel LFR performing comparably to LAR via a distinct coagulopathy-based mechanism. Lactate- and fibrinogen-based ratios were robust to inclusion of early deaths, whereas inflammation-based ratios were not, indicating that biomarker validity may depend on injury-to-death timing in pediatric trauma.

## 1. Introduction

Trauma remains one of the leading causes of admission to pediatric emergency departments and intensive care units, and continues to be a major contributor to childhood morbidity and mortality worldwide [1,2]. Trauma-related deaths in children are commonly described as occurring in early and late phases: early mortality is primarily attributable to hypoxia, hypovolemia, and severe traumatic brain injury, whereas late mortality is more often driven by the systemic inflammatory response syndrome (SIRS), multiple-organ dysfunction syndrome (MODS), sepsis, and acute respiratory distress syndrome [3,4]. Despite substantial advances in pediatric trauma systems, resuscitation strategies, and critical care management, outcomes related to post-traumatic systemic inflammation and organ dysfunction remain suboptimal [5,6].

Trauma triggers a rapid, complex inflammatory cascade driven by tissue injury, hypoperfusion, hypoxia, and cytokine release, which can progress to immune dysregulation and multi-organ dysfunction [7]. Early identification of children at risk for this trajectory is therefore critical for guiding intensive care management. Serum lactate, a marker of tissue hypoperfusion, and serum albumin, a negative acute-phase reactant reflecting inflammatory burden and physiologic reserve, have each been independently associated with adverse outcomes in critically ill children [8]. Their composite index, the lactate/albumin ratio (LAR), has demonstrated superior discriminatory performance compared with either marker alone in pediatric sepsis, cardiac arrest, and—more recently—trauma populations [8,9]. Large-scale adult trauma cohorts have further confirmed LAR as an independent predictor of in-hospital mortality and massive transfusion requirement, with discriminatory performance comparable to or exceeding established anatomical severity scores such as the injury severity score [10,11].

By the same physiological rationale, other inflammatory markers normalized to albumin may capture the balance between the inflammatory response and host reserve. The CRP/albumin ratio (CAR) has been explored as a prognostic marker in adult trauma and critical illness: an early study in trauma ICU patients demonstrated that CAR correlated with mortality on both the first and third day of admission [12], and CAR has more recently been confirmed as an independent risk factor for mortality in adults with moderate-to-severe traumatic brain injury, improving the discriminatory performance of prognostic models beyond the Glasgow Coma Scale alone [13]. Procalcitonin (PCT), unlike CRP, rises earlier after tissue injury and correlates more closely with the magnitude of trauma, and has shown independent predictive value for mortality in pediatric trauma cohorts [14,15]. Notably, a recent study in adult craniocerebral trauma demonstrated that the procalcitonin/albumin ratio (PAR) achieved excellent discriminatory performance for both injury severity and prognosis (AUC 0.79–0.80), performing comparably to CAR and outperforming either PCT or albumin alone [16]. However, to our knowledge, PAR has not been evaluated in pediatric trauma patients, nor has its serial trajectory during the early intensive care course been characterized in any trauma population.

Given that inflammatory activation (reflected by PCT and CRP) and tissue hypoperfusion (reflected by lactate) represent distinct, but interacting pathophysiological processes in trauma, we hypothesized that a composite index integrating all three markers relative to albumin might capture post-traumatic physiological derangement more comprehensively than any single ratio alone. We therefore *a priori* defined a novel composite biomarker, the triple inflammation–perfusion index (TIPI), calculated as the cube root of the product of PCT, CRP, and lactate, divided by albumin.

Beyond inflammation and perfusion, coagulopathy constitutes the third pillar of the “lethal triad”—together with acidosis and hypothermia—first described by Kashuk et al. in 1982 and now recognized as a central, mutually reinforcing driver of trauma-related mortality [17]. More recently, some authors have proposed extending this framework to a “lethal diamond” by incorporating hypocalcemia, or even a “lethal pentad” that further includes hypoxia and hyperglycemia. While these extended frameworks highlight the multifactorial nature of post-traumatic physiological failure, the present study focused specifically on the coagulopathy axis of the original triad [18]. Fibrinogen, unlike albumin, is typically a positive acute-phase reactant whose synthesis increases during systemic inflammation; however, in the setting of major hemorrhage, consumption, dilution from resuscitation fluids, and hyperfibrinolysis can overwhelm this compensatory rise, resulting in a paradoxical, rapid decline in circulating fibrinogen—the hallmark of trauma-induced coagulopathy, one of the three pillars of the lethal triad [17,19]. Low admission fibrinogen has been independently associated with mortality, massive transfusion, and organ dysfunction in adult trauma cohorts [20], and admission fibrinogen deficits have likewise been linked to mortality in pediatric traumatic brain injury [21]. A recent pediatric TBI study examined admission lactate and fibrinogen as separate predictors of outcome, but did not evaluate them as a combined ratio [22]. We therefore hypothesized that normalizing lactate and PCT to fibrinogen—rather than or in addition to albumin—might yield composite indices (the lactate/fibrinogen ratio [LFR] and PCT/fibrinogen ratio [PFR]) that capture the coagulopathic dimension of the lethal triad alongside hypoperfusion and inflammation, and that these fibrinogen-based ratios might perform comparably to or independently of their albumin-based counterparts.

The aim of this study was to evaluate the prognostic value of two novel fibrinogen-based composite ratios—the lactate/fibrinogen ratio (LFR) and PCT/fibrinogen ratio (PFR)—alongside the procalcitonin/albumin ratio (PAR) and TIPI for in-hospital mortality in pediatric trauma patients admitted to the pediatric intensive care unit (PICU). As secondary aims, we compared the discriminatory performance of these composite ratios with the lactate/albumin ratio (LAR), CRP/albumin ratio (CAR), and established severity scores (Pediatric Index of Mortality 3 [PIM3], Pediatric Risk of Mortality IV [PRISM4], injury severity score [ISS]), examined their correlation with severity scores and with one another, and evaluated whether their serial trajectories over the first 72 h provided additional prognostic information beyond single admission values.

## 2. Materials and Methods

This retrospective cohort study was conducted in the 54-bed Pediatric Intensive Care Unit (PICU) of İzmir City Hospital, a tertiary referral center providing advanced pediatric critical care services. Pediatric trauma patients admitted to the PICU between 27 October 2023 and 27 October 2025 were screened for eligibility.

Patients aged between 1 month and younger than 18 years admitted within 24 h of traumatic injury were eligible. Patients with known chronic organ failure, genetic or metabolic disorders, chronic inflammatory disease, or incomplete laboratory or clinical records were excluded. A total of 274 pediatric trauma patients met these criteria and were screened. Of these, 74 lacked a sufficiently complete clinical course to permit calculation of the serial (72 h) biomarker trajectories central to this study and were not included in the primary complete-trajectory cohort: 8 died within 72 h of admission, 44 were discharged from the PICU within 72 h, and 22 had a longer PICU stay, but incomplete follow-up laboratory sampling. After these exclusions, 200 patients met the eligibility criteria for the primary analysis. To address the possibility that this exclusion—which by definition removed the earliest deaths and the most rapidly recovering survivors—could have introduced selection bias into the admission-timepoint findings, the 74 excluded patients were also included in a separate sensitivity analysis restricted to admission (T0) data, described below. This yielded an unrestricted cohort of 274 patients (21 deaths) with complete T0 data, but without serial (T1–T3–discharge) follow-up.

Demographic (age, weight, height), trauma characteristics (mechanism—pedestrian struck by vehicle, motor vehicle occupant, motorcycle/bicycle/scooter, fall from height, drowning, penetrating trauma, other; anatomical region—head, thorax, abdomen, extremity, pelvis/spine, face), and clinical course data (respiratory support type and duration, inotropic support [vasoactive–inotropic score, VIS], transfusion requirements [packed red blood cells, fresh frozen plasma, platelets, cryoprecipitate], surgical intervention, number of organ dysfunctions, PICU length of stay) were retrieved from the hospital’s electronic medical record system.

Laboratory parameters—AST, LDH, CK, creatinine, uric acid, GFR, CRP, procalcitonin (PCT), lactate, albumin, fibrinogen (g/L), platelet count, hemoglobin, and neutrophil/lymphocyte ratio (NLR)—were recorded at four timepoints: admission (T0), 24 h (T1), 72 h (T3), and PICU discharge. Lactate was measured using point-of-care arterial or venous blood gas analyzers at the bedside, whereas all other laboratory parameters (CRP, PCT, albumin, fibrinogen, and complete blood count) were measured using standard assays in the hospital’s central laboratory, following routine clinical protocols throughout the study period. The CRP/albumin ratio (CAR), procalcitonin/albumin ratio (PAR), lactate/albumin ratio (LAR), lactate/fibrinogen ratio (LFR), and PCT/fibrinogen ratio (PFR) were calculated by dividing the respective marker by the simultaneously measured albumin or fibrinogen level at each timepoint. Fibrinogen was expressed in g/L (rather than mg/dL) for the calculation of LFR and PFR, consistent with the units conventionally used for albumin-based ratios in this study. A novel composite index, the triple inflammation–perfusion index (TIPI), was *a priori* defined as the cube root of the product of PCT, CRP, and lactate, divided by albumin: TIPI = ∛(PCT × CRP × lactate)/albumin. PAR and TIPI were prespecified as primary biomarkers of interest. LFR and PFR were developed as exploratory, hypothesis-generating extensions following observation of strong lactate- and fibrinogen-based associations in preliminary analyses, and their *p*-values should be interpreted with this exploratory framing in mind, without formal correction for multiple comparisons. Pediatric trauma score (PTS), Pediatric Index of Mortality 3 (PIM3), Pediatric Risk of Mortality IV (PRISM4), and injury severity score (ISS) were calculated at admission using standard criteria [23,24,25,26].

The primary outcome was in-hospital (PICU) mortality. Secondary outcomes were number of organ dysfunctions, PICU length of stay, and requirement for respiratory support, inotropic support, and blood product transfusion.

All statistical analyses were performed using IBM SPSS Statistics (version 23.0; IBM Corp., Armonk, NY, USA) and Python (version 3.12; SciPy, NumPy, and statsmodels libraries) for analyses not natively available in SPSS. Normality of continuous variables was assessed using the Shapiro–Wilk test, given the small number in the non-survivor group (*n* = 13). As the majority of variables violated the assumption of normal distribution, continuous variables are presented as medians (interquartile range [IQR]) and were compared between survivors and non-survivors using the Mann–Whitney U test. Categorical variables are presented as frequencies (percentages) and were compared using the χ^2^ test. Where more than 20% of cells had an expected count <5, the Fisher–Freeman–Halton exact test was used instead.

Receiver operating characteristic (ROC) curve analysis was performed to evaluate the discriminatory performance of PAR, LFR, PFR, TIPI, LAR, CAR, NLR, PTS, PIM3, PRISM4, and ISS for in-hospital mortality, and the area under the curve (AUC) with 95% confidence intervals was calculated. Optimal cut-off values were determined using the Youden index. Pairwise comparisons of AUCs between distinct, non-nested markers or scores (e.g., LFR vs. LAR) were performed using the DeLong test. Comparisons involving nested models (i.e., a marker or score alone versus the same marker or score combined with another predictor) were instead evaluated using the bootstrap-optimism-corrected ΔAUC procedure described below to avoid the optimism inherent in comparing nested models on the same data. To evaluate whether the early (24 h) and late (72 h) trajectories of PAR, LFR, PFR, TIPI, and LAR provided incremental discriminative value beyond their respective admission (T0) values, we compared a T0-only model with a model combining the T0 value and the corresponding absolute delta (Δ = value at 24 h or 72 h minus T0 value). Because these are nested models derived and evaluated within the same dataset, an initial within-sample DeLong comparison of the two AUCs would be expected to overstate incremental performance. This approach was therefore abandoned in favor of a bootstrap-based optimism-correction procedure applied directly to the AUC difference (ΔAUC) between the two models, following the general logic described by Harrell for single-model optimism correction. For each comparison and each of 1000 bootstrap resamples, both the T0-only and the T0-plus-delta models were refit on the resample. The ΔAUC was computed both on the resample itself (training performance) and by applying the same resample-fitted models to the original sample (test performance), and the difference between these two values (training minus test) was taken as the bootstrap estimate of optimism for that resample. The mean optimism across all resamples was subtracted from the same, fixed, full-sample apparent ΔAUC (unchanged across all resamples) to obtain an optimism-corrected ΔAUC, and a 95% percentile confidence interval was constructed from the distribution of (apparent ΔAUC − resample-specific optimism) across all resamples. The same procedure was applied to compare PRISM4 alone with PRISM4 combined with each biomarker.

Given the limited number of mortality events (*n* = 13), multivariable modeling was restricted to a maximum of two predictors to maintain an adequate events-per-variable ratio and reduce the risk of overfitting. Continuous composite biomarkers (PAR, LFR, PFR, TIPI, LAR, CAR, NLR) were log-transformed prior to regression modeling owing to marked right-skewness. Firth’s penalized maximum likelihood logistic regression was used throughout to reduce small-sample bias, and results are reported as odds ratios (ORs) with 95% confidence intervals [27]. Internal validation of all regression models was performed by bootstrap resampling (B = 1000 for discrimination and Brier score, B = 500 for calibration slope), and optimism-corrected AUC, Brier score, and calibration slope are reported alongside apparent (uncorrected) values. Spearman correlation analysis was used to evaluate the association of composite ratios with severity scores and with one another.

To evaluate whether the 72 h data-completeness requirement introduced selection bias into the admission-timepoint findings, a sensitivity analysis was performed in the unrestricted cohort of 274 patients described above (200 primary-analysis patients plus the 74 patients excluded for incomplete serial follow-up) using only T0 (admission) data. ROC/AUC analysis, univariable Firth logistic regression, and Spearman correlation with severity scores were repeated for all composite ratios and severity scores in this unrestricted cohort following the same methods described above. Because serial (T1–T3–discharge) data were unavailable for the 74 additionally included patients by definition, trajectory-based analyses could not be and were not repeated in this cohort.

A two-sided *p*-value < 0.05 was considered statistically significant throughout.

## 3. Results

Of 200 pediatric trauma patients admitted to the PICU during the study period, 13 (6.5%) died during their PICU stay. The remaining 187 (93.5%) survived to discharge. The median age was 145 months (IQR 46.5–192) among survivors and 188 months (IQR 167–189) among non-survivors, and age did not differ significantly between groups (*p* = 0.161), nor did weight (*p* = 0.136), height (*p* = 0.284), or sex (72 female [36.0%], 128 male [64.0%] overall; Fisher’s exact *p* = 1.000).

Pedestrian struck by vehicle (36.5%) and fall from height (35.0%) were the most common trauma mechanisms, followed by penetrating trauma and motor vehicle/bicycle/scooter injuries (7.5% each) and motor vehicle occupant injury (6.0%). Head injury was the most frequent anatomical region affected (65.5%), followed by thoracic (12.0%) and abdominal (11.5%) injuries. Although non-survivors showed a numerically higher proportion of the pedestrian-struck-by-vehicle mechanism (69.2% vs. 34.2% in survivors; adjusted residual +2.5), the overall association between trauma mechanism and mortality did not reach statistical significance (Fisher’s exact *p* = 0.075), and trauma region was not associated with mortality (*p* = 0.840).

Non-survivors had significantly lower Glasgow Coma Scale scores (median 3 [IQR 3–4] vs. 15 [8–15], *p* < 0.001; overall cohort median 14 [8–15]) and significantly lower (more severe) pediatric trauma scores (2 [−1–4] vs. 7 [3–9], *p* < 0.001) than survivors. Non-survivors were significantly more likely to require invasive mechanical ventilation (100% vs. 32.6%, *p* < 0.001), inotropic support (30.8% vs. 3.2%, *p* = 0.002), and blood product transfusion (84.6% vs. 30.5%, *p* < 0.001), and were significantly more likely to develop multi-organ dysfunction (92.3% vs. 7%, *p* < 0.001). Surgical intervention did not differ between groups (*p* = 0.565). PICU length of stay did not differ significantly between groups (8 days [IQR 3–10] in non-survivors vs. 5 [4–10] in survivors, *p* = 0.932). As expected, all three severity scores were markedly higher in non-survivors: PIM3 5.1 (IQR 2.2–84.8) versus 1.1 (0.7–2.3), PRISM4 96 (37–99) versus 9 (5–18), and ISS 12 (9–13) versus 6 (5–9) (all *p* < 0.001). Full baseline and comparative characteristics are presented in Table 1.

At admission (T0), non-survivors had significantly higher lactate (4.1 mmol/L [IQR 3.1–6.0] vs. 1.7 [1.1–2.95], *p* < 0.001), significantly lower albumin (3.36 g/dL [2.94–4.00] vs. 4.17 [3.71–4.44], *p* = 0.009), and significantly lower fibrinogen (1.58 g/L [IQR 1.09–2.00] vs. 2.32 [1.90–2.90], *p* = 0.003) than survivors. Among the composite ratios, LAR was markedly higher in non-survivors (1.20 [0.96–1.60] vs. 0.44 [0.275–0.735], *p* < 0.001), as were PAR (0.932 [0.069–1.115] vs. 0.08 [0.022–0.318], *p* = 0.036) and TIPI (1.292 [0.545–2.583] vs. 0.344 [0.167–0.903], *p* = 0.005). The two novel fibrinogen-based ratios were likewise significantly elevated in non-survivors: LFR was markedly higher (2.77 [IQR 2.30–3.17] vs. 0.81 [0.43–1.24], *p* < 0.001), as was PFR (1.10 [0.21–2.56] vs. 0.13 [0.04–0.55], *p* = 0.008). Notably, neither of PAR’s individual components reached significance at this timepoint when considered alone—CRP (14.0 [2.0–21.0] vs. 4.0 [1.0–21.15], *p* = 0.160) and PCT (2.74 ng/mL [0.23–3.85] vs. 0.28 [0.09–1.06], *p* = 0.051)—nor did CAR (4.87 [0.68–6.19] vs. 1.00 [0.27–5.63], *p* = 0.108). NLR did not differ between groups at T0 (8.50 [5.37–10.40] vs. 8.28 [3.33–14.05], *p* = 0.984) or at most subsequent timepoints, reaching significance only at 72 h (*p* = 0.013). Organ-function markers (AST, LDH, CK, creatinine, uric acid, GFR) were already significantly altered in non-survivors at admission (all *p* ≤ 0.006), whereas hemoglobin (*p* = 0.465) and platelet count (*p* = 0.158) were not.

ROC curve analysis showed that LFR achieved the highest point-estimate discriminatory performance among all composite ratios evaluated (AUC 0.848, 95% CI 0.683–0.962), numerically exceeding LAR (0.829, 95% CI 0.686–0.972), followed by TIPI (0.731), PFR (0.719, 95% CI 0.533–0.874), and PAR (0.674); CAR (0.631) and NLR (0.502) remained non-discriminatory (Table 2, Figure 1). PRISM4 had the highest overall discriminatory performance (AUC 0.924, 95% CI 0.846–1.000), followed by PIM3 (0.837), ISS (0.827), and PTS (0.803). DeLong testing showed that LFR’s discrimination did not differ significantly from LAR (*p* = 0.399), TIPI (*p* = 0.113), or PFR (*p* = 0.100), nor did PFR differ significantly from PAR (*p* = 0.074, a borderline trend favoring PFR) or TIPI (*p* = 0.792). LFR showed a non-significant trend toward lower discrimination than PRISM4 (*p* = 0.060). As with PAR and TIPI, adding LFR to PRISM4 did not significantly improve discrimination over PRISM4 alone (bootstrap-corrected ΔAUC −0.003, 95% CI −0.036 to 0.025).

Given the shared lactate numerator between LFR and LAR, we assessed their redundancy directly: combining LFR with LAR provided no significant incremental discrimination over LAR alone (bootstrap-corrected ΔAUC −0.033, 95% CI −0.241 to 0.056), and the two ratios were strongly correlated (Spearman ρ = 0.873, *p* < 0.001). Similarly, PFR was very strongly correlated with PAR (ρ = 0.972, *p* < 0.001), reflecting their shared PCT numerator. In contrast, LFR and PFR were only weakly correlated with each other (ρ = 0.196) and with TIPI (ρ = 0.183 for LFR).

Given the limited number of mortality events (*n* = 13), multivariable models were restricted to two predictors. In univariable Firth penalized logistic regression, log-transformed LFR was among the strongest predictors identified in this study (OR 5.22, 95% CI 2.38–11.44, *p* < 0.001), exceeding the effect size observed for LAR (OR 4.66, 2.15–10.12, *p* < 0.001); PIM3 (OR 2.84, *p* < 0.001), PRISM4 (OR 1.06, *p* < 0.001), PTS (OR 1.31, *p* = 0.001), ISS (OR 1.19, *p* = 0.002), TIPI (OR 2.14, *p* = 0.003), PFR (OR 1.74, 95% CI 1.19–2.54, *p* = 0.004), and PAR (OR 1.43, *p* = 0.021) were each independently associated with mortality, whereas CAR (*p* = 0.099) and NLR (*p* = 0.696) were not (Table 3). When PAR, TIPI, or LFR were added to PRISM4 in two-predictor models, PRISM4 remained the dominant predictor in all cases (all *p* < 0.001) while the biomarker term lost statistical significance (PAR *p* = 0.353, TIPI *p* = 0.964, LFR *p* = 0.282), mirroring the bootstrap-corrected findings above.

Bootstrap internal validation (B = 1000) showed minimal optimism for all single-predictor models, including the two fibrinogen-based ratios (LFR: apparent AUC 0.848, corrected 0.848, optimism −0.001; PFR: apparent 0.719, corrected 0.719, optimism 0.000), indicating internally consistent discrimination comparable to that observed for PRISM4, LAR, PAR, and TIPI individually, though external generalizability remains untested. Two-predictor models combining PRISM4 with a biomarker showed greater optimism (0.008–0.022), consistent with limited incremental value and a modest overfitting risk when a second predictor was added in this small-event dataset (Table 4a, Figure 2).

Spearman correlation analysis showed that PAR, TIPI, LAR, CAR, LFR, and PFR each correlated significantly with PRISM4, ISS, and PTS (all *p* ≤ 0.012), supporting their biological plausibility as markers of overall injury severity; correlation with PIM3 did not reach significance for any of the six ratios (Table 5). PAR and TIPI were highly correlated with one another (ρ = 0.879) and with CAR (TIPI-CAR ρ = 0.850, PAR-CAR ρ = 0.577), reflecting their shared inflammatory components; PFR was likewise highly correlated with PAR (ρ = 0.972) and TIPI (ρ = 0.846). LAR showed only weak-to-moderate correlation with the albumin-based inflammatory ratios (ρ = 0.04–0.34), but very strong correlation with LFR (ρ = 0.873), its fibrinogen-based counterpart.

Examining the serial trajectory of the composite ratios revealed two distinct patterns. Across the four sampling timepoints, PAR, TIPI, and CAR were significantly higher in non-survivors from 24 h onward, while LAR remained the most consistently discriminative marker throughout (*p* < 0.001 at all timepoints; Figure 3).

Because nested models—the admission value alone versus the admission value plus trajectory—were both derived and evaluated within the same dataset, we assessed incremental discriminative value using a bootstrap-based optimism-correction procedure applied to the AUC difference (ΔAUC) itself, rather than a within-sample DeLong comparison (Table 4b; see Section 2). Using this procedure, only the 24 h trajectory of PAR showed a 95% confidence interval that excluded zero (ΔAUC 0.172, 95% CI 0.035 to 0.275). Given the limited number of events and the absence of formal correction for multiple comparisons across the ten trajectory tests performed, this should be regarded as a possible, exploratory signal rather than a confirmed effect. No other comparison—including TIPI at 24 or 72 h, LAR at either timepoint, or LFR/PFR at either timepoint—yielded a confidence interval excluding zero (Table 4b). Combining PRISM4 with PAR, TIPI, or LFR likewise showed no evidence of incremental value (all 95% CIs spanning zero and narrowly centered near zero; Table 4c). Although LFR and PFR did not show robust incremental value when their trajectories were combined with the admission value, their 24 and 72 h absolute changes each differed significantly between survivors and non-survivors when tested individually (Mann–Whitney U; LFR: Δ24 h *p* < 0.001, Δ72 h *p* = 0.004; PFR: Δ24 h *p* = 0.010, Δ72 h *p* = 0.015). Given the wide confidence intervals inherent to this analysis with only 13 mortality events, these results should be interpreted as largely inconclusive regarding the incremental value of serial biomarker trajectories, with the possible exception of PAR’s 24 h trajectory, which warrants further evaluation in a larger cohort.

To assess whether the requirement of complete 72 h serial data introduced selection bias into the admission-timepoint findings above—given that this criterion necessarily excluded both the earliest deaths and the most rapidly recovering survivors—a sensitivity analysis was performed in the unrestricted cohort of 274 patients (21 deaths, 7.7%), comprising the primary 200-patient cohort plus the 74 patients excluded for incomplete serial follow-up (8 early deaths, 44 early discharges, and 22 patients with a longer stay but incomplete follow-up sampling; see Section 2).

In this unrestricted cohort, LFR and LAR retained essentially unchanged discriminatory performance (LFR: AUC 0.851, 95% CI 0.752–0.929; LAR: AUC 0.833, 95% CI 0.727–0.920), closely matching their performance in the primary cohort, and remained independently associated with mortality in univariable Firth regression (LFR: OR 4.81, 95% CI 2.54–9.10, *p* < 0.001; LAR: OR 4.98, 95% CI 2.55–9.74, *p* < 0.001). In contrast, the discriminatory performance of PAR, TIPI, and PFR was substantially attenuated and no longer statistically significant in the unrestricted cohort (PAR: AUC 0.545, *p* = 0.494; TIPI: AUC 0.594, *p* = 0.153; PFR: AUC 0.583, *p* = 0.204), as was their association with mortality in Firth regression (all *p* ≥ 0.053; Table 6). CAR and NLR remained non-discriminatory in both cohorts. All six ratios remained significantly correlated with PRISM4, ISS, and PTS in the unrestricted cohort, indicating that their association with overall injury severity was preserved even where their specific discrimination of mortality was not. These findings indicate that the lactate-based ratios (LAR, LFR) are robust to the inclusion of the earliest deaths and most rapidly recovering survivors excluded from the primary analysis, whereas the apparent discriminatory value of the inflammation-based ratios (PAR, TIPI, PFR) in the primary cohort may be specific to the subpopulation surviving to at least 72 h, and should be interpreted with corresponding caution.

## 4. Discussion

In this retrospective cohort of 200 pediatric trauma patients admitted to a tertiary PICU, we found that two lactate-based composite ratios achieved the strongest single-timepoint discrimination for in-hospital mortality among all biomarkers evaluated. The established lactate/albumin ratio (LAR) and our novel lactate/fibrinogen ratio (LFR) performed similarly well. LFR numerically exceeded LAR (AUC 0.848 vs. 0.829), although this difference did not reach statistical significance (DeLong *p* = 0.399). The procalcitonin/albumin ratio (PAR), our composite triple inflammation–perfusion index (TIPI), and the novel PCT/fibrinogen ratio (PFR) were also independently associated with mortality. Notably, neither raw PCT, raw CRP, nor the CRP/albumin ratio (CAR) reached significance individually at admission. To our knowledge, this is the first study to evaluate fibrinogen-normalized composite ratios, grounded in the classic “lethal triad” concept of trauma-induced coagulopathy, alongside albumin-normalized ratios in a pediatric trauma population [17,19].

The strong correlation between LFR and LAR (Spearman ρ = 0.873) and between PFR and PAR (ρ = 0.972) indicates that for a given numerator, the choice of denominator captures largely overlapping prognostic information. This finding is consistent with our observation that combining LFR with LAR failed to provide significant incremental discrimination over LAR alone (bootstrap-corrected ΔAUC −0.033, 95% CI −0.241 to 0.056). The overlap is biologically plausible. Although fibrinogen and albumin differ fundamentally in their acute-phase behavior—fibrinogen typically rises during systemic inflammation, whereas albumin falls—both can ultimately decline in severe trauma: fibrinogen through consumption, dilution, and hyperfibrinolysis, and albumin through suppressed synthesis and capillary leak. This convergent, if mechanistically distinct, depletion may explain why LFR and LAR nonetheless captured overlapping prognostic information despite reflecting different underlying processes [19]. Nonetheless, LFR achieved the numerically highest point-estimate performance of any biomarker evaluated in this study, including LAR. Combined with its distinct conceptual grounding in coagulopathy rather than generalized nutritional and inflammatory reserve, this suggests that fibrinogen may be at least as valid a choice as albumin for normalizing lactate-based composite indices in trauma. This may be of practical relevance: international trauma resuscitation guidelines already prioritize early fibrinogen assessment and repletion as a first-line target in bleeding trauma patients [28], whereas albumin is typically obtained as part of a standard metabolic panel without a comparable urgency protocol. A fibrinogen-based ratio may therefore integrate more naturally into existing point-of-care coagulation workflows than an albumin-based one independently of any difference in prognostic accuracy between the two.

We had hypothesized that LFR and PFR might behave similarly to PAR and TIPI with respect to serial trajectory. Instead, the fibrinogen-based ratios followed a pattern resembling LAR. Their 24 and 72 h absolute changes differed significantly between survivors and non-survivors when considered individually (see Section 3), but neither trajectory provided incremental discrimination beyond the admission value once assessed using the bootstrap-optimism-corrected ΔAUC procedure described above (all 95% CIs spanning zero; Table 4b). As with LAR, we interpret this as a ceiling effect: both LFR and PFR already achieved strong admission discrimination, leaving comparatively little room for the trajectory to add further prognostic information. PAR and TIPI, by contrast, had more modest baseline performance and correspondingly greater room for improvement. Visually, the PFR trajectory nonetheless showed a transient rise in non-survivors at 24 h reminiscent of PAR and TIPI (Figure 3), raising the possibility that this pattern could reach significance in a larger cohort. Given the wide confidence intervals inherent to a 13-event cohort, this observation should be considered hypothesis-generating rather than a definitive negative finding.

The biological plausibility of all six composite ratios evaluated is supported by our correlation analysis. PAR, TIPI, LAR, CAR, LFR, and PFR were each significantly associated with PRISM4, ISS, and PTS, indicating that they track overall injury severity rather than representing isolated, unrelated signals. Notably, none of the six ratios correlated significantly with PIM3. Because this pattern held consistently across the entire panel of composite biomarkers, it likely reflects a methodological difference between scoring systems rather than a marker-specific idiosyncrasy. PIM3 is calculated from physiological variables recorded at a single timepoint close to PICU arrival, whereas PRISM4 uses the worst values recorded over the first 24 h—a window that more closely parallels the cumulative laboratory alteration captured by our composite ratios. This distinction may explain why PRISM4—but not PIM3—correlated consistently with all six biomarkers evaluated.

Adding LFR to PRISM4 did not significantly improve discrimination over PRISM4 alone (Table 4c), mirroring the pattern already reported above for PAR and TIPI. We interpret this as a ceiling effect: PRISM4 achieved near-maximal discrimination in this cohort (AUC 0.924), leaving little room for any additional biomarker to contribute measurable improvement. Bingöl and Altınsoy (2026) observed a similar pattern when combining LAR with PRISM III [9]. The correlation between LAR and PRISM4 (ρ = 0.407), and to a lesser extent between LFR and PRISM4 (ρ = 0.330), suggests that a substantial portion of the lactate-based ratios’ prognostic information is already captured within PRISM4′s composite physiological scoring. This does not diminish the clinical utility of these biomarkers as simple, rapidly available laboratory tools. LFR in particular achieved discrimination statistically indistinguishable from LAR using a coagulation parameter that is often drawn as part of routine trauma admission panels. Rather, it tempers claims of superiority over existing multivariable severity-scoring systems in favor of a more modest claim of complementarity.

Using a rigorous bootstrap-based optimism-correction procedure to address the risk of overstating incremental performance from nested models evaluated within the same dataset—a methodological safeguard adopted following peer review—we found limited evidence that serial biomarker trajectories over the first 72 h added discriminative information beyond single admission values. Only the 24 h trajectory of PAR showed a confidence interval excluding zero (Table 4b). No other comparison, including TIPI, reached this threshold. A possible biological rationale for this modest signal is that procalcitonin, the numerator of PAR, is not corrected by simple hemodynamic resuscitation or blood product administration, unlike lactate- or fibrinogen-related coagulopathy, and instead reflects an ongoing inflammatory and catabolic process over the first days after injury [14,15]; however, given the wide confidence intervals inherent to a 13-event cohort, this finding should be regarded as hypothesis-generating rather than confirmatory. The remaining comparisons—including TIPI, LAR, LFR, and PFR at both timepoints, and all PRISM4-combination models—showed confidence intervals spanning zero and should be considered inconclusive rather than evidence for or against a true effect. This more conservative conclusion differs from our initial, less rigorously validated analysis, and underscores the importance of appropriately accounting for optimism when evaluating nested prediction models in small-event datasets—a point we return to in the summary of limitations.

A related concern is that the 72 h data-completeness requirement by construction excluded both the earliest deaths and the most rapidly recovering survivors from the primary analysis—a form of selection bias that could plausibly have distorted even the admission-timepoint (T0) findings reported above. To address this directly, we repeated the T0 analyses (ROC/AUC, univariable Firth regression, and correlation with severity scores) in an unrestricted cohort of 274 patients, combining the primary 200-patient cohort with the 74 patients not included in the primary complete-trajectory cohort due to incomplete serial follow-up (8 who died within 72 h, 44 discharged within 72 h, and 22 with incomplete follow-up sampling despite a longer stay). This sensitivity analysis yielded a striking and biologically informative divergence: LFR and LAR retained essentially unchanged discriminatory performance (LFR: AUC 0.851; LAR: AUC 0.833) and remained strongly significant predictors (both *p* < 0.001; Table 6), whereas PAR, TIPI, and PFR lost statistical significance entirely (Firth OR *p* ≥ 0.053; AUC-based Mann–Whitney *p* ≥ 0.153). We interpret this divergence as reflecting the differing kinetics of the underlying biological processes rather than a spurious artifact: lactate and fibrinogen respond to hemorrhagic shock and coagulopathy within minutes to hours, whereas procalcitonin and CRP require hours to days to rise even in critically ill patients. The eight patients who died within 72 h of admission—most within the first one to two days—may therefore not yet have mounted a detectable procalcitonin or CRP response at the time of admission sampling, despite being the most physiologically compromised patients in the cohort, thereby diluting the discriminative performance of the PCT/CRP-based ratios when these early deaths are included. This finding both strengthens confidence in LAR and LFR as robust, timing-independent predictors of mortality, and appropriately tempers the admission-timepoint findings for PAR, TIPI, and PFR reported in the primary analysis, suggesting that their apparent prognostic value may be relatively specific to patients surviving beyond the earliest, most rapidly fatal phase of injury rather than generalizable across the full spectrum of pediatric trauma mortality.

Several findings warrant further comment. The neutrophil-to-lymphocyte ratio showed essentially no discriminatory value at admission (AUC 0.502, *p* = 0.984) and reached significance only transiently at 72 h (*p* = 0.013), in contrast to previous pediatric trauma studies reporting more consistent associations between NLR and mortality [9,14]. This discrepancy may reflect differences in injury mechanism distribution, timing of sampling, or the comparatively small number of events in our cohort limiting statistical power to detect a modest effect. Hemoglobin and platelet counts did not differ between survivors and non-survivors despite significant derangement in coagulation-related organ markers and admission fibrinogen. This pattern is consistent with the interpretation, offered in prior pediatric trauma cohorts [9], that early trauma-associated coagulopathy is driven primarily by consumption of coagulation factors, reflected specifically in the fibrinogen decline observed here, and by hepatic dysfunction rather than by frank anemia or thrombocytopenia. Although the mechanism of injury was not significantly associated with mortality overall (Fisher’s exact *p* = 0.075), post-hoc adjusted residuals suggested a disproportionate representation of pedestrian-vehicle collisions among non-survivors, consistent with the high kinetic energy transfer characteristic of this mechanism. Given the limited event count, this observation should be regarded as hypothesis-generating.

This study has several important limitations. The retrospective, single-center design limits generalizability and precluded systematic recording of finer-grained mechanism subcategories (e.g., helmet use in bicycle/motorcycle injuries, fall height), and the modest overall mortality rate (6.5%, *n* = 13 deaths) substantially constrained statistical power for multivariable modeling and subgroup analyses. In addition, our inclusion criteria required a clinical course extending to at least 72 h to permit calculation of complete serial biomarker trajectories, meaning that patients who died very early or who were discharged rapidly before this window were not represented in the analytic cohort. This selection criterion means our trajectory-based findings (necessarily limited to the 200-patient cohort with complete serial data) apply specifically to patients with a clinical course extending to at least 72 h. As reported above, a sensitivity analysis extending the admission-timepoint analyses to the full unrestricted cohort of 274 patients confirmed that LAR and LFR remain robust predictors regardless of this restriction, whereas the admission-timepoint findings for PAR, TIPI, and PFR should be interpreted as applying specifically to the 72 h-survival subpopulation rather than the full spectrum of pediatric trauma severity. We restricted multivariable models to two predictors and applied Firth’s penalized likelihood method to reduce small-sample bias [27], but residual overfitting cannot be entirely excluded despite bootstrap internal validation showing minimal optimism for single-predictor models, including LFR and PFR. Composite ratios were log-transformed prior to regression modeling to satisfy linearity assumptions. While this is standard practice for skewed laboratory ratios, it complicates direct clinical interpretation of odds ratios and should be considered when translating these findings into bedside cut-off values. Although LFR achieved the numerically highest point-estimate discrimination in this study, its close correlation with LAR means our findings should not be interpreted as establishing fibrinogen-based ratios as categorically superior to albumin-based ratios. They instead demonstrate comparable performance with a distinct and complementary conceptual rationale. Finally, external validation in an independent, larger, and ideally multicenter pediatric trauma cohort is required before any of these composite ratios can be recommended for clinical decision-making, including assessment of whether LFR retains its numerical advantage over LAR with greater statistical power.

## 5. Conclusions

In this cohort of pediatric trauma patients admitted to the PICU, lactate-based composite ratios achieved the strongest single-timepoint discrimination for in-hospital mortality. Because PRISM4 alone achieved the highest overall discrimination and no biomarker significantly improved on it in combination, these composite ratios should be regarded not as replacements for multivariable severity scores such as PRISM4, but as simpler, single-value alternatives that may be useful when rapid bedside assessment or full physiological scoring is impractical. Both the established LAR and the novel fibrinogen-based LFR performed comparably well, despite reflecting distinct physiological processes: LAR capturing tissue hypoperfusion (linked to the acidosis component of the lethal triad) and reduced protein synthesis capacity versus LFR capturing coagulopathy specifically, the triad’s third component. The procalcitonin/albumin ratio, PCT/fibrinogen ratio, and TIPI were also independently associated with mortality in the primary cohort despite weaker individual components; however, a sensitivity analysis in an unrestricted cohort that additionally included early deaths and rapidly discharged survivors showed that this association was not robust, in contrast to the consistently strong performance of LAR and LFR across both cohorts. This divergence suggests that the prognostic value of lactate- and fibrinogen-based ratios is largely independent of the timing of death, whereas that of inflammation-based ratios may be specific to patients surviving beyond the earliest, most rapidly fatal phase of injury. Using a rigorous bootstrap-based optimism-correction procedure to guard against overstating the performance of nested models evaluated on the same data, we found only limited and largely inconclusive evidence for the incremental value of serial biomarker trajectories, with a modest, hypothesis-generating signal restricted to the 24 h trajectory of PAR. This distinction highlights both the promise and the methodological caution required when evaluating serial biomarker trajectories in small-event pediatric cohorts. If validated in larger, prospective cohorts, these findings suggest that a uniform serial testing strategy across all biomarker classes may not be optimal; however, because the discriminatory value of PCT-based ratios was itself not robust once the earliest deaths were included, any future protocol emphasizing repeat PCT-based measurement would need to be validated specifically within this high-risk subpopulation—precisely the patients for whom such a protocol would matter most—before being recommended. These findings should be considered exploratory and hypothesis-generating: the small number of mortality events limits the precision of effect-size estimates, and prospective, externally validated studies in larger, multicenter cohorts are needed before any threshold values reported here are applied in clinical practice.

## Figures and Tables

**Figure 1:**
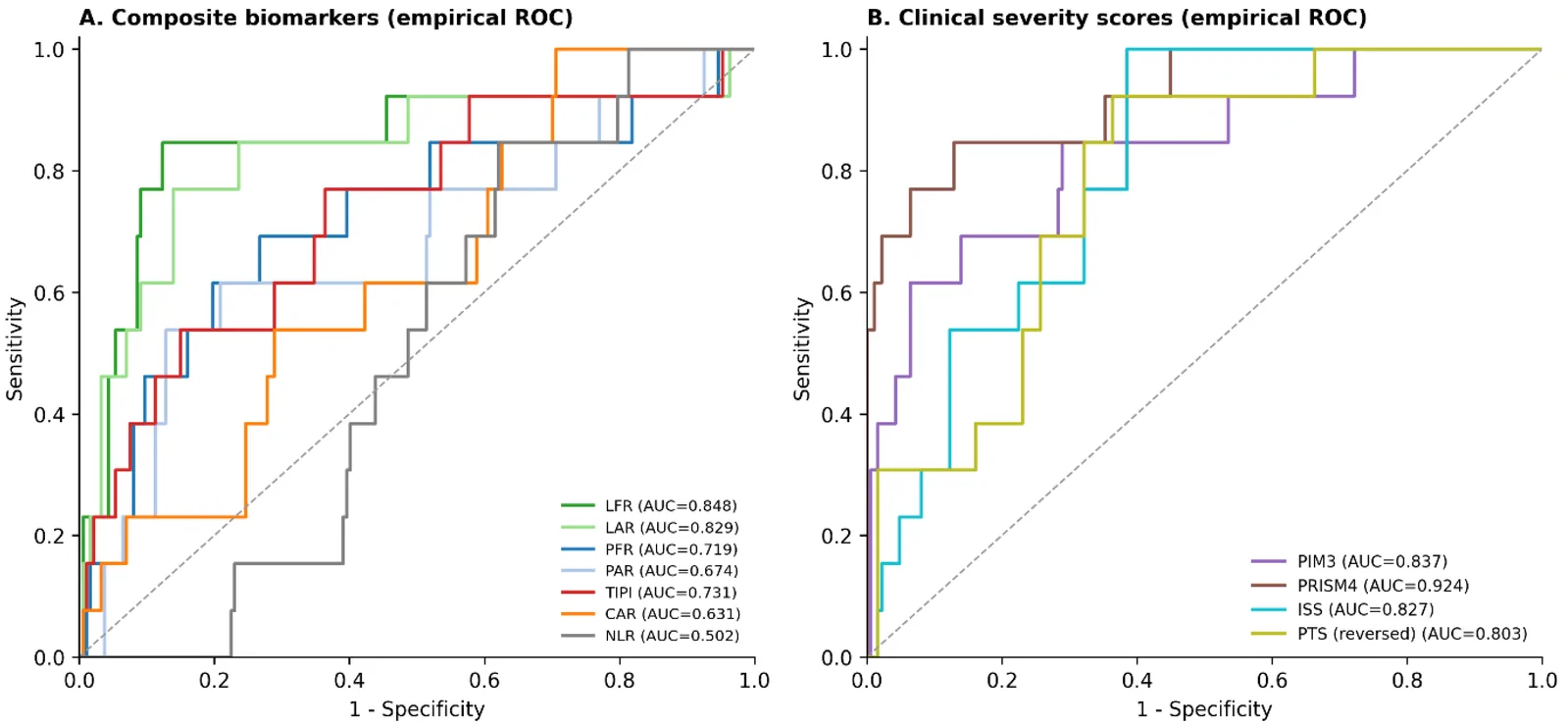
Receiver operating characteristic (ROC) curves for in-hospital mortality prediction. (**A**) Composite biomarkers: lactate/fibrinogen ratio (LFR), lactate/albumin ratio (LAR), PCT/fibrinogen ratio (PFR), procalcitonin/albumin ratio (PAR), triple inflammation–perfusion index (TIPI), CRP/albumin ratio (CAR), and neutrophil/lymphocyte ratio (NLR), measured at admission (T0). (**B**) Established clinical severity scores: Pediatric Index of Mortality 3 (PIM3), Pediatric Risk of Mortality IV (PRISM4), injury severity score (ISS), and pediatric trauma score (PTS, reverse-coded so that higher values indicate greater severity, consistent with panel A). The diagonal dashed line represents the line of no discrimination (AUC = 0.5). Curves represent the empirical (non-smoothed) receiver operating characteristic function. Given the limited number of mortality events (*n* = 13), curves are step functions rather than continuous lines, reflecting the discrete resolution of the underlying data.

**Figure 2:**
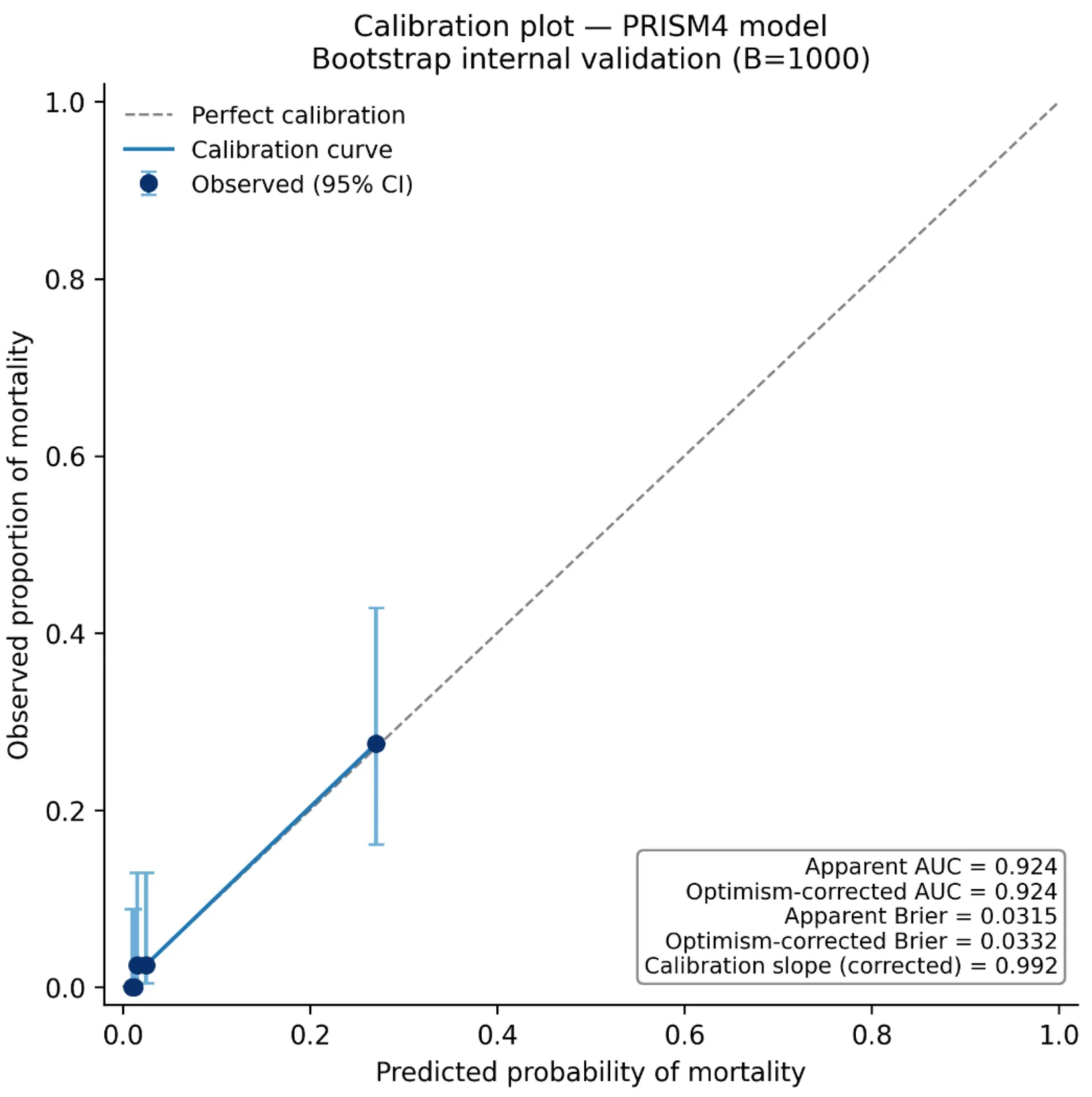
Calibration plot for the PRISM4 mortality prediction model, with bootstrap internal validation (B = 1000). Patients were grouped into quintiles of predicted mortality risk. Observed proportions of mortality (dark circles, with 95% Wilson confidence intervals) are plotted against the mean predicted probability within each quintile. The gray dashed line represents perfect calibration (predicted = observed). Apparent and optimism-corrected discrimination (AUC), Brier score, and calibration slope are shown in the inset.

**Figure 3:**
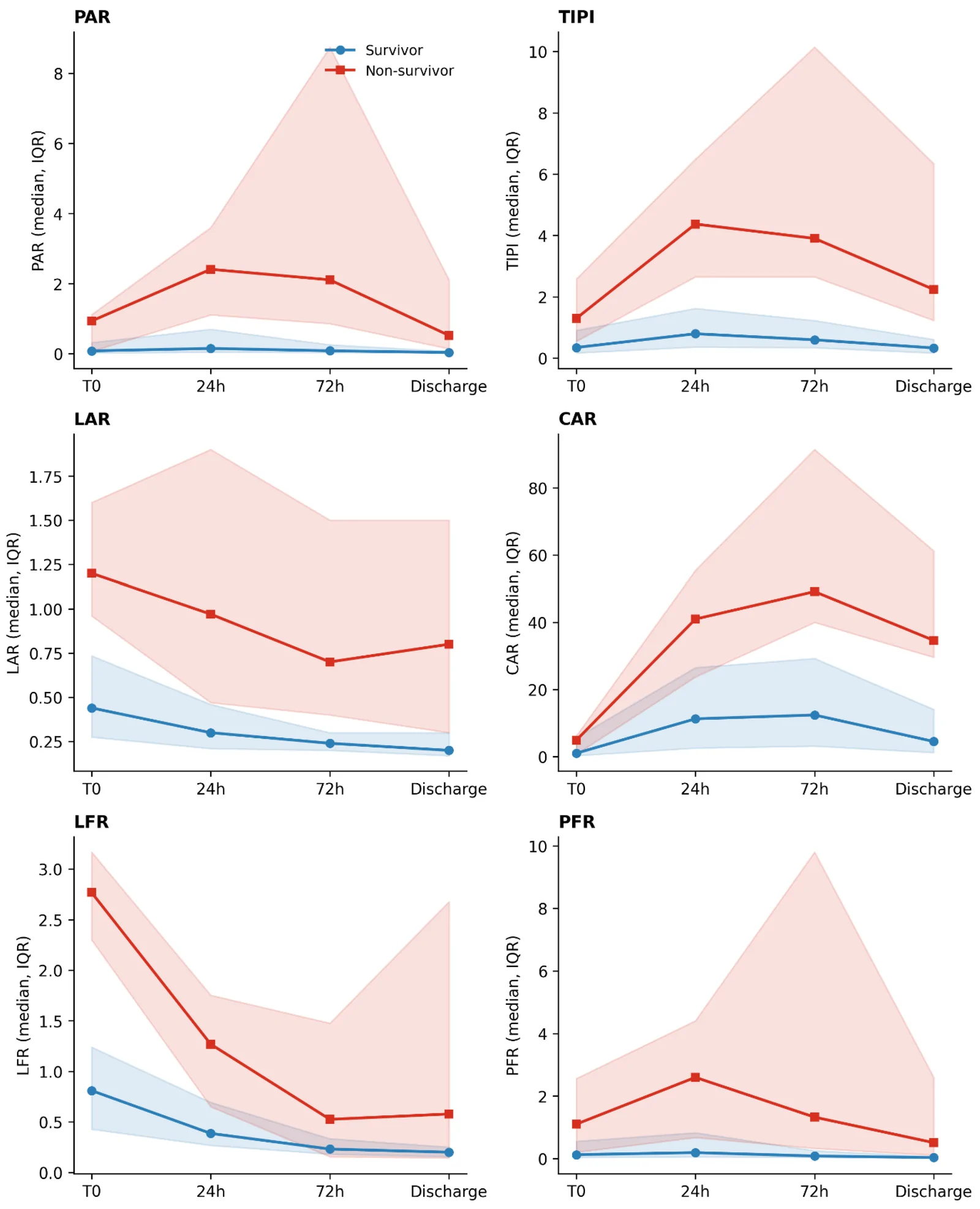
Serial trajectory of composite biomarkers from admission to PICU discharge by survival status. Median values (points) and interquartile range (shaded bands) of the procalcitonin/albumin ratio (PAR), triple inflammation–perfusion index (TIPI), lactate/albumin ratio (LAR), CRP/albumin ratio (CAR), lactate/fibrinogen ratio (LFR), and PCT/fibrinogen ratio (PFR) at admission (T0), 24 h, 72 h, and PICU discharge, stratified by survivors (blue) and non-survivors (red). Note the visually divergent trajectory shape for PAR, TIPI, and PFR—rising in non-survivors before declining, while remaining comparatively stable in survivors—in contrast to LAR and LFR, whose trajectories decline in parallel in both groups; however, only PAR’s 24 h trajectory reached statistical significance in bootstrap-corrected analysis (see Section 3 and Table 4b).

**Table 1:** Baseline characteristics and comparison of survivors and non-survivors.

Characteristic		Overall (*n* = 200)	Survivors (*n* = 187)	Non-Survivors (*n* = 13)	*p*
Demographics					
Age, months		156 (50.3–192)	145 (46.5–192)	188 (167–189)	0.161
Sex, *n* (%)	Male	128 (64.0)	120 (64.2)	8 (61.5)	1.000
	Female	72 (36.0)	67 (35.8)	5 (38.5)	
Trauma characteristics, *n* (%)					
Mechanism	Pedestrian struck by vehicle	73 (36.5)	64 (34.2)	9 (69.2)	0.075
	Motor vehicle occupant	12 (6.0)	10 (5.3)	2 (15.4)	
	Motor/bicycle/scooter	15 (7.5)	14 (7.5)	1 (7.7)	
	Fall from height	70 (35.0)	69 (36.9)	1 (7.7)	
	Drowning	7 (3.5)	7 (3.7)	0 (0)	
	Penetrating trauma	15 (7.5)	15 (8.0)	0 (0)	
	Other	8 (4.0)	8 (4.3)	0 (0)	
Region	Head	131 (65.5)	121 (64.7)	10 (76.9)	0.840
	Thoracic	24 (12.0)	23 (12.3)	1 (7.7)	
	Abdominal	23 (11.5)	21 (11.2)	2 (15.4)	
	Extremity	18 (9.0)	18 (9.6)	0 (0)	
	Pelvis/spine	4 (2.0)	4 (2.1)	0 (0)	
Clinical severity and course					
GCS		14 (8–15)	15 (8–15)	3 (3–4)	<0.001
PTS		7 (2.25–9)	7 (3–9)	2 (−1–4)	<0.001
PTS category	Fatal	12 (6.0)	8 (4.3)	4 (30.8)	<0.001
	Life-threatening	60 (30.0)	52 (27.8)	8 (61.5)	
	Potentially life-threatening	64 (32.0)	63 (33.7)	1 (7.7)	
	Minor trauma	64 (32.0)	64 (34.2)	0 (0)	
PIM3		1.1 (0.7–2.4)	1.1 (0.7–2.3)	5.1 (2.2–84.8)	<0.001
PRISM4		9 (5–20.25)	9 (5–18)	96 (37–99)	<0.001
ISS		7 (5–9.75)	6 (5–9)	12 (9–13)	<0.001
Respiratory support: IMV		74 (37)	61 (32.6)	13 (100)	<0.001
Inotropic support required		10 (5.0)	6 (3.2)	4 (30.8)	0.002
Transfusion required		68 (34.0)	57 (30.5)	11 (84.6)	<0.001
Surgical intervention		87 (43.5)	80 (42.8)	7 (53.8)	0.565
Multi-organ dysfunction		25 (12.5)	13 (7)	12 (92.3)	<0.001
PICU length of stay, days		5 (4–10)	5 (4–10)	8 (3–10)	0.932
T0 laboratory findings					
Lactate, mmol/L		1.8 (1.1–3.1)	1.7 (1.1–2.95)	4.1 (3.1–6.0)	<0.001
CRP, mg/L		4.0 (1.0–21.23)	4.0 (1.0–21.15)	14.0 (2.0–21.0)	0.160
PCT, ng/mL		0.30 (0.09–1.34)	0.28 (0.09–1.06)	2.74 (0.23–3.85)	0.051
Albumin, g/dL		4.14 (3.60–4.42)	4.17 (3.71–4.44)	3.36 (2.94–4.00)	0.009
Fibrinogen, g/L		2.23 (1.81–2.90)	2.32 (1.90–2.90)	1.58 (1.09–2.00)	0.003
CAR		1.02 (0.28–6.17)	1.00 (0.27–5.63)	4.87 (0.68–6.19)	0.108
PAR		0.081 (0.022–0.361)	0.08 (0.022–0.318)	0.932 (0.069–1.115)	0.036
LAR		0.455 (0.29–0.80)	0.44 (0.275–0.735)	1.20 (0.96–1.60)	<0.001
LFR		0.84 (0.44–1.35)	0.81 (0.43–1.24)	2.77 (2.30–3.17)	<0.001
PFR		0.14 (0.04–0.62)	0.13 (0.04–0.55)	1.10 (0.21–2.56)	0.008
NLR		8.29 (3.40–13.90)	8.28 (3.33–14.05)	8.50 (5.37–10.40)	0.984
TIPI		0.369 (0.170–0.930)	0.344 (0.167–0.903)	1.292 (0.545–2.583)	0.005

Data are medians (IQR) or *n* (%). Categorical variables compared with χ^2^ or Fisher’s exact test; continuous variables with Mann–Whitney U test. Abbreviations: CAR, CRP/albumin ratio; GCS, Glasgow Coma Scale; ISS, injury severity score; IMV, invasive mechanical ventilation; LAR, lactate/albumin ratio; LFR, lactate/fibrinogen ratio; NLR, neutrophil/lymphocyte ratio; PAR, procalcitonin/albumin ratio; PICU, pediatric intensive care unit; PIM3, Pediatric Index of Mortality 3; PFR, procalcitonin/fibrinogen ratio; PRISM4, Pediatric Risk of Mortality IV; PTS, pediatric trauma score; TIPI, triple inflammation–perfusion index.

**Table 2:** Predictive performance of markers for in-hospital mortality (ROC analysis, T0).

Marker	AUC	95% CI	*p*	Cut-Off	Sensitivity	Specificity	Youden Index
PRISM4	0.924	(0.846, 1.000)	<0.001	≥26.5	0.846	0.872	0.718
LFR	0.848	(0.683, 0.962)	<0.001	≥2.02	0.846	0.877	0.723
PIM3	0.837	(0.716, 0.957)	<0.001	≥1.95	0.846	0.711	0.557
LAR	0.829	(0.686, 0.972)	<0.001	≥0.955	0.769	0.861	0.630
ISS	0.827	(0.747, 0.907)	<0.001	≥7.5	1.000	0.615	0.615
PTS	0.803 *	(0.706, 0.900)	<0.001	≤5.5	0.923	0.636	0.559
TIPI	0.731	(0.579, 0.884)	0.005	≥0.542	0.769	0.636	0.405
PFR	0.719	(0.533, 0.874)	0.008	≥0.48	0.692	0.733	0.425
PAR	0.674	(0.506, 0.841)	0.037	≥0.856	0.538	0.872	0.410
CAR	0.631	(0.492, 0.771)	0.114	—	—	—	—
NLR	0.502	(0.390, 0.613)	0.984	—	—	—	—

* PTS analyzed as reverse-coded (−PTS) given its inverse relationship with severity. Table shows results in the original (clinical) direction. AUC, area under the curve. Cut-off values were derived using the Youden index for descriptive purposes only and should not be interpreted as validated clinical decision thresholds given the small number of events. Dashes (—) indicate that no cut-off value was derived because the corresponding AUC was not statistically significant.

**Table 3:** Firth penalized logistic regression—univariable and two-predictor models.

Model	Variable	OR	95% CI	*p*
Univariable	LFR (log)	5.22	(2.38, 11.44)	<0.001
	LAR (log)	4.66	(2.15, 10.12)	<0.001
	PIM3 (log)	2.84	(1.80, 4.48)	<0.001
	PRISM4	1.06	(1.04, 1.09)	<0.001
	PTS (reversed)	1.31	(1.11, 1.55)	0.001
	ISS	1.19	(1.07, 1.33)	0.002
	TIPI (log)	2.14	(1.29, 3.55)	0.003
	PFR (log)	1.74	(1.19, 2.54)	0.004
	PAR (log)	1.43	(1.06, 1.95)	0.021
	CAR (log)	1.30	(0.95, 1.77)	0.099
	NLR (log)	1.10	(0.68, 1.80)	0.696
PRISM4 + PAR	PAR (log)	0.80	(0.51, 1.28)	0.353
	PRISM4	1.07	(1.04, 1.10)	<0.001
PRISM4 + TIPI	TIPI (log)	0.99	(0.52, 1.87)	0.964
	PRISM4	1.06	(1.04, 1.09)	<0.001
PRISM4 + LFR	LFR (log)	1.58	(0.69, 3.62)	0.282
	PRISM4	1.06	(1.03, 1.08)	<0.001

Continuous composite ratios were log-transformed due to right-skewness. Multivariable models limited to two predictors given *n* = 13 mortality events.

**Table 4:** Bootstrap internal validation (B = 1000/500)—optimism-corrected performance.

(a) Single-predictor models						
Model	AUC (apparent)	AUC (corrected)	Optimism	Brier (apparent)	Brier (corrected)	Calibration slope
PRISM4 alone	0.924	0.924	~0.000	0.0315	0.0332	0.992
LFR (log) alone	0.848	0.848	−0.001	0.0493	0.0517	1.018
LAR (log) alone	0.829	0.831	−0.002	0.0522	0.0543	1.027
PFR (log) alone	0.719	0.719	0.000	0.0585	0.0602	1.628 *
PAR (log) alone	0.674	0.668	0.005	0.0592	0.0606	1.112
TIPI (log) alone	0.731	0.735	−0.003	0.0569	0.0585	1.578 *
PRISM4 + PAR	0.906	0.888	0.018	0.0310	0.0343	0.956
PRISM4 + TIPI	0.925	0.903	0.022	0.0315	0.0349	0.955
PRISM4 + LFR	0.898	0.890	0.008	0.0299	0.0328	0.943
(b) Incremental value of 24/72 h trajectory beyond admission value (ΔAUC)						
Comparison	ΔAUC (apparent)		ΔAUC (corrected)		95% CI	
PAR_0_ + Δ24 h	0.173		0.172		(0.035, 0.275)	
PAR_0_ + Δ72 h	0.139		0.123		(−0.126, 0.311)	
TIPI_0_ + Δ24 h	0.162		0.150		(−0.046, 0.263)	
TIPI_0_ + Δ72 h	0.192		0.174		(−0.055, 0.287)	
LAR_0_ + Δ24 h	−0.023		−0.040		(−0.214, 0.072)	
LAR_0_ + Δ72 h	0.048		0.044		(−0.052, 0.105)	
LFR_0_ + Δ24 h	−0.013		−0.015		(−0.064, 0.041)	
LFR_0_ + Δ72 h	−0.008		−0.010		(−0.057, 0.031)	
PFR_0_ + Δ24 h	0.111		0.104		(−0.061, 0.216)	
PFR_0_ + Δ72 h	−0.041		−0.058		(−0.314, 0.161)	
(c) Incremental value of adding a biomarker to PRISM4						
Comparison	ΔAUC (apparent)		ΔAUC (corrected)		95% CI	
PRISM4 + PAR	0.002		0.000		(−0.019, 0.011)	
PRISM4 + TIPI	−0.003		−0.012		(−0.064, 0.009)	
PRISM4 + LFR	0.000		−0.003		(−0.036, 0.025)	

* Instability likely reflects sparse-event bootstrap resamples for this single-predictor model—interpret cautiously. Bootstrap-corrected ΔAUC and 95% CI derived using a Harrell-style optimism-correction procedure (B = 1000): both nested models were refit on each resample, and the mean difference between training-sample and original-sample ΔAUC (optimism) was subtracted from the apparent ΔAUC. CIs constructed as percentiles of (apparent ΔAUC − resample optimism) across all resamples.

**Table 5:** Spearman correlations of composite ratios with severity scores and with one another.

(a) Correlation with severity scores					
	PIM3	PRISM4	ISS	PTS	
PAR	ρ = 0.104, *p* = 0.141	ρ = 0.293, *p* < 0.001	ρ = 0.421, *p* < 0.001	ρ = −0.401, *p* < 0.001	
TIPI	ρ = 0.104, *p* = 0.143	ρ = 0.357, *p* < 0.001	ρ = 0.450, *p* < 0.001	ρ = −0.380, *p* < 0.001	
LAR	ρ = 0.036, *p* = 0.609	ρ = 0.407, *p* < 0.001	ρ = 0.236, *p* = 0.001	ρ = −0.226, *p* = 0.001	
CAR	ρ = 0.082, *p* = 0.248	ρ = 0.223, *p* = 0.001	ρ = 0.326, *p* < 0.001	ρ = −0.227, *p* = 0.001	
LFR	ρ = 0.009, *p* = 0.904	ρ = 0.330, *p* < 0.001	ρ = 0.177, *p* = 0.012	ρ = −0.192, *p* = 0.006	
PFR	ρ = 0.112, *p* = 0.114	ρ = 0.299, *p* < 0.001	ρ = 0.416, *p* < 0.001	ρ = −0.417, *p* < 0.001	
(b) Correlation among composite ratios					
	PAR	TIPI	LAR	CAR	LFR
TIPI	0.879	—	—	—	—
LAR	0.209	0.336	—	—	—
CAR	0.577	0.850	0.042	—	—
LFR	0.079	0.183	0.873	−0.073	—
PFR	0.972	0.846	0.226	0.537	0.196

Values in panel (b) are Spearman’s ρ. All correlations were statistically significant (*p* < 0.01) except PAR–LFR (*p* = 0.265), LAR–CAR (*p* = 0.555), and CAR–LFR (*p* = 0.303). Dashes (—) indicate cells that are not applicable, i.e., self-correlations and the duplicated half of the symmetric correlation matrix.

**Table 6:** Sensitivity analysis (*n* = 274 unrestricted cohort).

Marker	AUC, Primary Cohort (*n* = 200)	AUC, Unrestricted Cohort (*n* = 274)	95% CI	AUC, *p*	Firth OR (Unrestricted)	95% CI	OR *p*
LFR	0.848	0.851	0.752–0.929	<0.001	4.81	2.54–9.10	<0.001
LAR	0.829	0.833	0.727–0.920	<0.001	4.98	2.55–9.74	<0.001
PFR	0.719	0.583	0.429–0.729	0.204	1.22	0.96–1.56	0.106
PAR	0.674	0.545	0.395–0.694	0.494	1.18	0.91–1.53	0.220
TIPI	0.731	0.594	0.447–0.744	0.153	1.48	1.00–2.19	0.053
CAR	0.631	0.495	0.355–0.642	0.936	1.04	0.81–1.33	0.772
NLR	0.502	0.473	0.374–0.569	0.684	0.98	0.67–1.43	0.920
PIM3	0.837	0.878	0.782–0.958	<0.001	3.56	2.35–5.40	<0.001
PRISM4	0.924	0.891	0.821–0.952	<0.001	1.05	1.04–1.07	<0.001
ISS	0.827	0.826	0.746–0.899	<0.001	1.08	1.05–1.12	<0.001
PTS	0.803	0.823	0.755–0.885	<0.001	1.36	1.19–1.56	<0.001

Unrestricted cohort: 274 patients (21 deaths), combining the primary 200-patient cohort with 74 patients not included in the primary complete-trajectory cohort due to incomplete 72 h serial follow-up. Composite ratios log-transformed for Firth regression. Continuous biomarker ORs reflect per-unit increase in log-transformed value.

## Data Availability

The data presented in this study are available on request from the corresponding author due to privacy restrictions relating to pediatric patient data.

## Abbreviations

The following abbreviations are used in this manuscript.

AST	aspartate aminotransferase
AUC	area under the (ROC) curve
CAR	CRP/albumin ratio
CI	confidence interval
CK	creatine kinase
CRP	C-reactive protein
GCS	Glasgow Coma Scale
GFR	glomerular filtration rate
HGB	hemoglobin
ICU	intensive care unit
IMV	invasive mechanical ventilation
IQR	interquartile range
ISS	injury severity score
LAR	lactate/albumin ratio
LDH	lactate dehydrogenase
LFR	lactate/fibrinogen ratio
MODS	multiple organ dysfunction syndrome
NLR	neutrophil/lymphocyte ratio
OR	odds ratio
PAR	procalcitonin/albumin ratio
PCT	procalcitonin
PFR	procalcitonin/fibrinogen ratio
PICU	pediatric intensive care unit
PIM3	Pediatric Index of Mortality 3
PLT	platelet count
PRISM4	Pediatric Risk of Mortality IV
PTS	pediatric trauma score
ROC	receiver operating characteristic
SIRS	systemic inflammatory response syndrome
TIPI	triple inflammation–perfusion index
VIS	vasoactive–inotropic score
